# Reversible Sepsis-Induced Myocardial Dysfunction in Term Pregnancy: A Case Report of a Rare Medical Condition

**DOI:** 10.7759/cureus.106877

**Published:** 2026-04-12

**Authors:** Bedih Balkan, Nazli Gonulduru, Ebru Kaya, Gulseren Yilmaz

**Affiliations:** 1 Intensive Care Unit, Kanuni Sultan Suleyman Training and Research Hospital, University of Health Sciences, Istanbul, TUR; 2 Anesthesiology and Reanimation, Kanuni Sultan Suleyman Training and Research Hospital, University of Health Sciences, Istanbul, TUR

**Keywords:** acute sepsis, heart disease in pregnancy, reversible heart failure, sepsis and septic shock, sepsis-induced myocardial dysfunction

## Abstract

Sepsis-induced myocardial dysfunction (SIMD) is a reversible heart failure that occurs during sepsis or septic shock. Although rare in pregnancy, its clinical presentation can be very similar to other peripartum heart complications, making diagnosis difficult. We report a previously healthy 28-year-old primigravida woman at 38 weeks of gestation who presented with an acute onset of dyspnea (New York Heart Association Class IV). The patient had developed upper respiratory tract symptoms approximately seven days prior to admission, including fever and malaise, which progressively worsened. A nonspecific, blanching maculopapular rash on the trunk and extremities was noted on physical examination. An emergency cesarean section was performed due to clinical deterioration of the mother and fetal distress. Postnatal echocardiography showed a left ventricular ejection fraction (LVEF) of 38% and global hypokinesia, but no apical ballooning or regional wall motion abnormalities. Radiological evidence of pneumonia, in combination with elevated inflammatory markers (high-sensitivity C-reactive protein, procalcitonin, leukocytosis) and increased cardiac biomarkers (troponin, N-terminal pro-B-type natriuretic peptide), supported the diagnosis of SIMD. Targeted antibiotic therapy, cautious fluid management, and low-dose norepinephrine were administered. Rapid improvement in heart function was observed after infection control, with LVEF returning to 55% before discharge. In cases of acute heart failure associated with sepsis during pregnancy, SIMD should always be considered in the differential diagnosis. Early detection, appropriate infection treatment, and prompt hemodynamic support can greatly improve maternal and neonatal outcomes.

## Introduction

Pregnancy is a complex physiological process that causes extensive cardiovascular, hormonal, and immunological adaptations. These changes are essential for fetoplacental circulation and preparing the body for delivery; however, modifications in the maternal immune response may increase susceptibility to infections [1-3]. Increased immune tolerance, higher cardiac preload and stroke volume, and decreased systemic vascular resistance may predispose pregnant women to rapid progression and severe symptoms of sepsis [2,3]. Cardiac dysfunction during sepsis is a key factor in determining prognosis. Sepsis-induced myocardial dysfunction (SIMD) is characterized by temporary but significant myocardial contractile depression and is linked with high mortality [4,5]. Proposed mechanisms include cytokine-driven inflammation, mitochondrial dysfunction, altered calcium regulation, and nitric oxide-induced myocardial depression [5,6]. Recent studies have shown that approximately 40%-50% of patients with severe sepsis or septic shock develop myocardial dysfunction. Early recognition and prompt initiation of hemodynamic support significantly reduce mortality [4-6]. Here, we report a case of reversible myocardial dysfunction secondary to pneumonia-induced sepsis in a term pregnancy. This case emphasizes the importance of distinguishing SIMD from peripartum cardiomyopathy (PPCM) and Takotsubo cardiomyopathy (TCM), which share overlapping clinical features but require different management approaches. Accurate differentiation is crucial for optimizing treatment strategies and enhancing maternal prognosis.

## Case presentation

A 28-year-old nulliparous woman at 38 weeks of gestation arrived at the emergency department with sudden and rapidly worsening shortness of breath, orthopnea, and malaise. She had no previous history of heart disease, high blood pressure, or diabetes. Her pregnancy had been uneventful until one week prior to admission, when she developed symptoms of an upper respiratory infection.

On admission, vital signs showed significant hemodynamic instability: blood pressure 80/60 mmHg, heart rate 130 bpm, respiratory rate 28 breaths/minute, body temperature 38.2°C, and oxygen saturation 86% on room air, and physical examination revealed jugular venous distention, bilateral basal crackles, and a nonspecific blanching maculopapular rash on the trunk and extremities; in the presence of markedly elevated inflammatory markers (C-reactive protein (CRP), procalcitonin, and leukocytosis), these findings were suggestive of a systemic inflammatory response consistent with bacterial infection.

Due to worsening respiratory distress and fetal bradycardia, an emergency cesarean section was performed under general anesthesia, resulting in the delivery of a healthy neonate. In the immediate postoperative period, the patient’s condition further deteriorated, and arterial blood gas analysis revealed a pH of 7.30, a partial pressure of carbon dioxide of 30 mmHg, and an arterial oxygen partial pressure (PaO₂) of 88 mmHg, corresponding to a PaO₂/fractional inspired oxygen ratio of 220, consistent with acute lung injury. This clinical deterioration necessitated endotracheal intubation and admission to the intensive care unit (ICU) for advanced hemodynamic and respiratory support.

Laboratory results showed severe systemic inflammation: CRP was 280 mg/L, procalcitonin was 15 ng/mL, and leukocytes were 18,000/µL. Cardiac biomarkers were elevated, with troponin I at 0.8 ng/mL and N-terminal pro-B-type natriuretic peptide at 600 pg/mL. Serum lactate was 4.8 mmol/L, indicating tissue hypoperfusion, and creatinine was mildly elevated at 1.5 mg/dL. Arterial blood gas analysis revealed metabolic acidosis with a pH of 7.21. Blood and sputum cultures grew *Klebsiella pneumoniae*. The Pneumonia Severity Index was calculated as 108 (Class IV), indicating high-risk pneumonia. The laboratory parameters at different time points are summarized in Table 1.

**Table 1:** Laboratory parameters at different time points during the clinical course NT-proBNP: N-terminal pro-B-type natriuretic peptide

Laboratory parameters	Reference range	Day 1	Day 3	Day 12
C-reactive protein	<5 mg/L	280 mg/L	72 mg/L	3.8 mg/L
Procalcitonin	<0.5 ng/mL	15 ng/mL	4 ng/mL	0.21 ng/mL
Leukocytes	3.8-10 × 10³/μL	18 × 10³/μL	12 × 10³/μL	6.7 × 10³/μL
Troponin I	<0.014 ng/mL	0.8 ng/mL	0.31 ng/mL	0.007 ng/mL
NT-proBNP	<125 pg/mL	600 pg/mL	211 pg/mL	80 pg/mL
Lactate	0.5-2 mmol/L	4.8 mmol/L	0.8 mmol/L	0.5 mmol/L
Creatinine	0.50-0.90 mg/dL	1.5 mg/dL	1.2 mg/dL	0.59 mg/dL
pH	7.35-7.45	7.21	7.31	7.40

**Figure 1:**
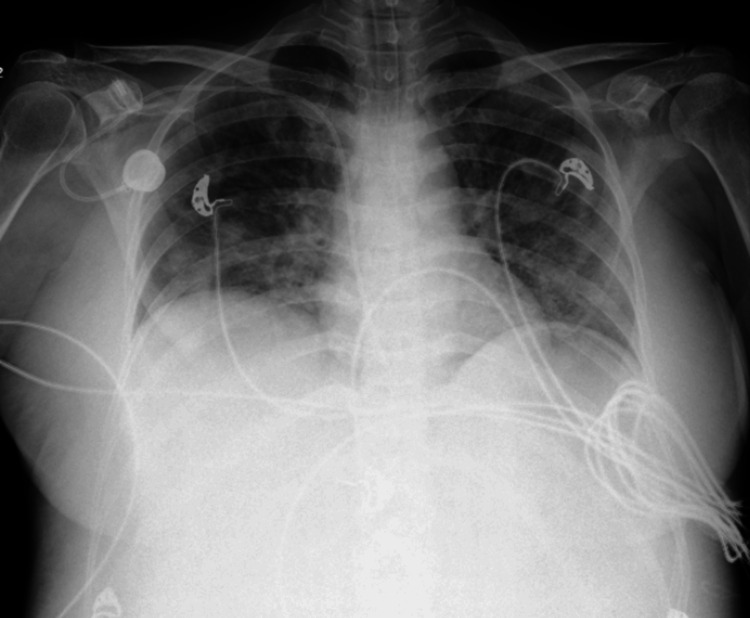
Chest X-ray demonstrating right lower lobe consolidation

**Figure 2:**
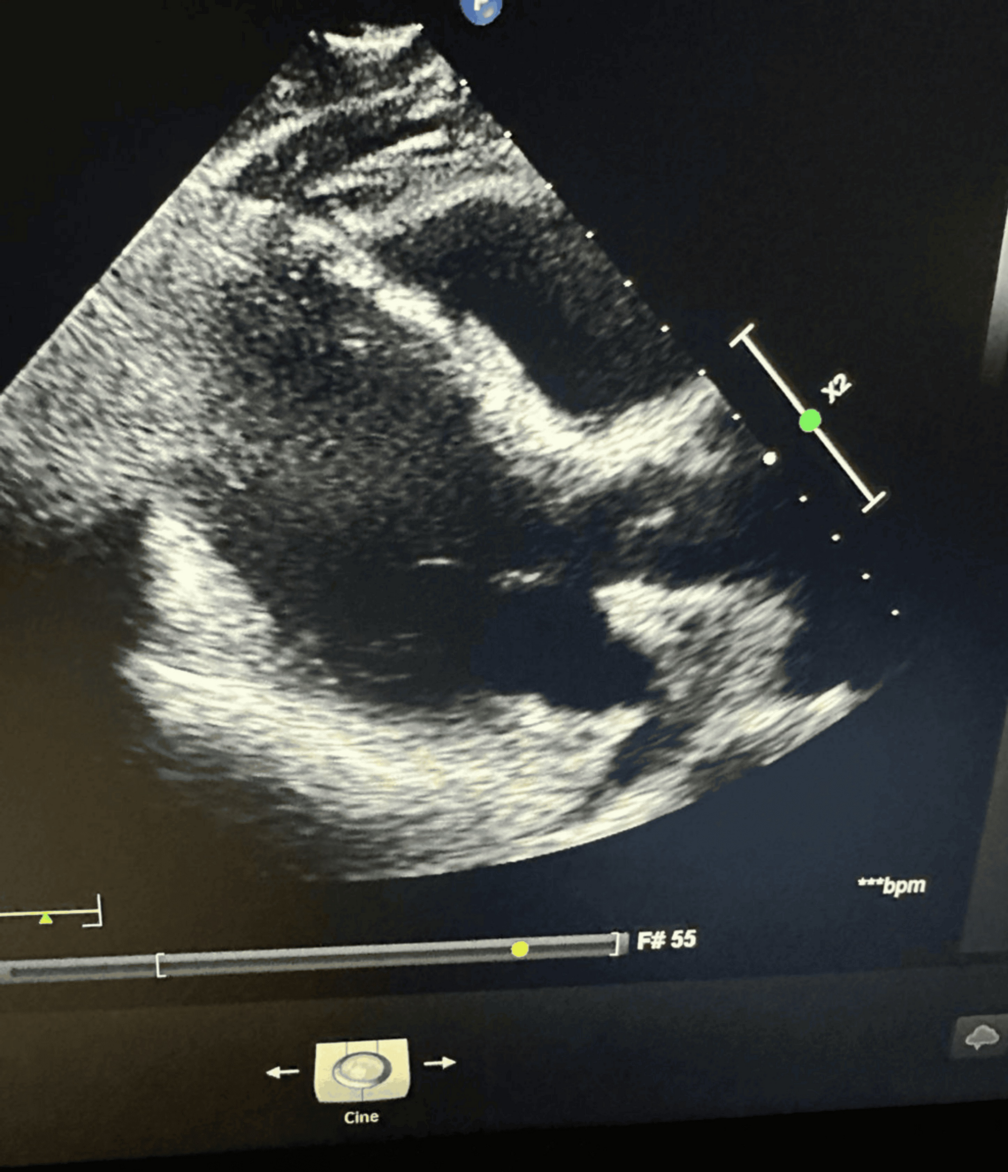
Transthoracic echocardiographic image demonstrating global hypokinesia with a left ventricular ejection fraction of 38% (Simpson’s biplane method)

Chest radiography revealed consolidation in the right lower lobe, indicating pneumonia, as shown in Figure 1. Transthoracic echocardiography (TTE) showed global hypokinesia with a left ventricular ejection fraction (LVEF) of 38% (Simpson’s biplane method), as shown in Figure 2. Although pericardial thickening was suspected in a single echocardiographic view, it was not consistently observed throughout the full examination. The absence of apical ballooning or regional wall motion abnormalities ruled out TCM.

The differential diagnosis included SIMD, PPCM, and TCM. The presence of diffuse hypokinesia, elevated inflammatory markers, and active infection favored SIMD. Given the moderate troponin elevation and lack of ischemic symptoms, coronary angiography was not indicated.

A multidisciplinary ICU team obtained microbiological samples, including blood, urine, and tracheal aspirate cultures, upon admission to the ICU, and initiated empirical broad-spectrum antibiotic therapy with meropenem (1 g every eight hours, IV) and vancomycin (1 g every 12 hours, IV) immediately thereafter. The patient did not receive antibiotic therapy intraoperatively. Fluid resuscitation was performed cautiously, and low-dose norepinephrine infusion was titrated to maintain a mean arterial pressure of at least 65 mmHg. Mechanical ventilation was continued using lung-protective strategies. Stress ulcer prophylaxis was provided with intravenous pantoprazole (40 mg once daily), and venous thromboembolism prophylaxis was administered using subcutaneous enoxaparin sodium (4,000 anti-Xa IU once daily).

By day 3, inflammatory markers and hemodynamics had significantly improved, and lactate levels had normalized. The patient was successfully extubated on day 8 and transitioned to spontaneous breathing. By day 12, she was hemodynamically stable and transferred to the obstetrics ward.

**Figure 3:**
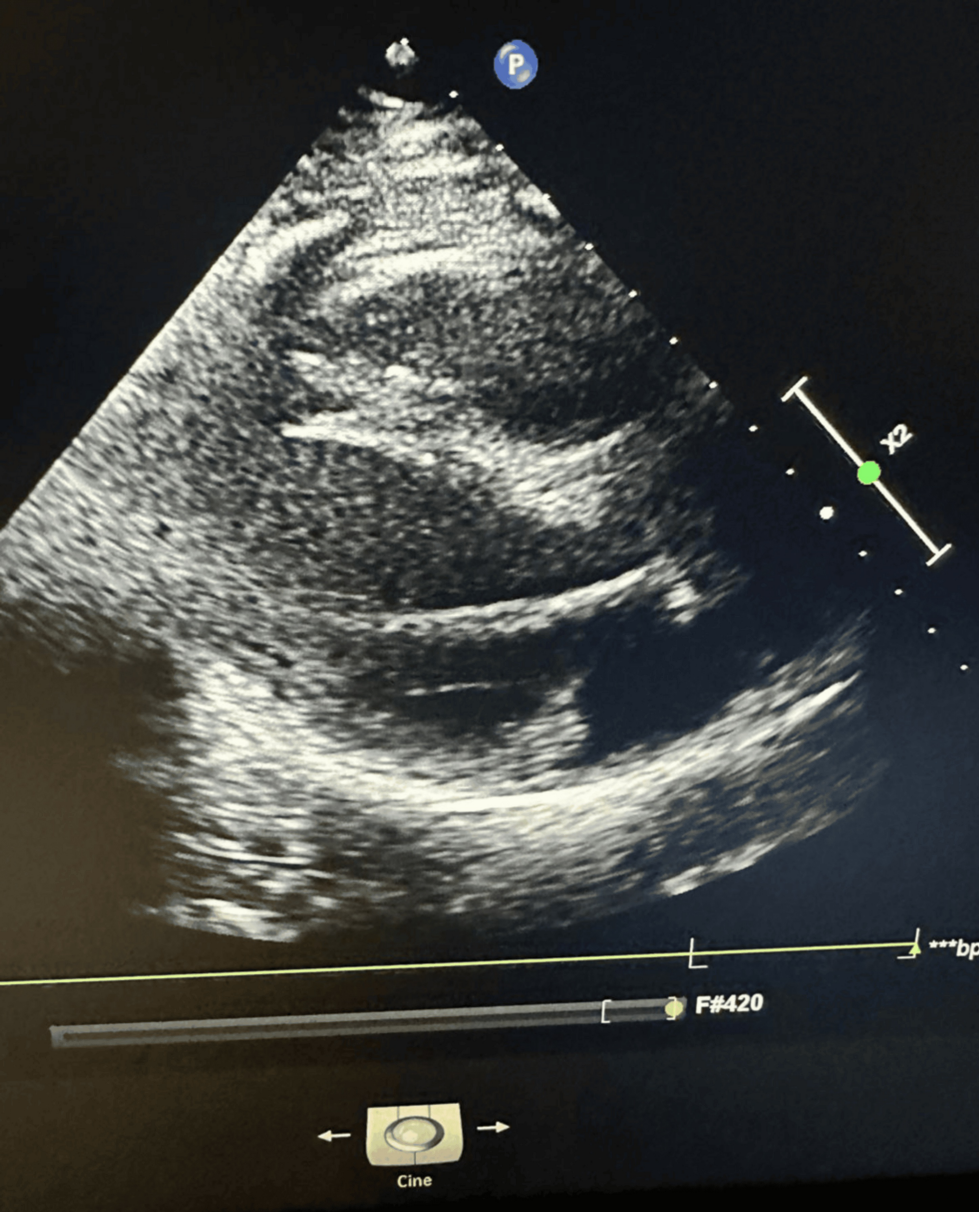
Follow-up transthoracic echocardiography showing normalized left ventricular systolic function (55%) with complete resolution of wall motion abnormalities

Guideline-directed medical therapy for acute decompensated heart failure was not initiated, as myocardial dysfunction was considered secondary to SIMD. Management was primarily focused on infection control and hemodynamic stabilization. Follow-up TTE before discharge showed normalization of left ventricular function (LVEF 55%), as demonstrated in Figure 3, further confirming the reversible nature of myocardial dysfunction. This case shows that SIMD in late pregnancy can resemble PPCM and TCM clinically, but with prompt diagnosis and focused treatment, full cardiac recovery is possible. Written informed consent was obtained from the patient for publication of clinical details and accompanying images.

## Discussion

SIMD is a common yet frequently underrecognized complication of sepsis and septic shock, marked by temporary, widespread ventricular contractile impairment [7]. Its pathophysiology involves several interacting mechanisms, including excessive proinflammatory cytokine release (cytokine storm), increased nitric oxide production, mitochondrial energy failure, and disturbances in calcium handling [8]. These processes cause reversible myocardial depression without lasting structural damage. Usually, cardiac function recovers within 7-10 days [9].

Clinically, SIMD presents with hypotension, tachycardia, increased requirement for inotropic support, and echocardiographic evidence of global hypokinesia [10]. The absence of coronary artery disease, moderate troponin elevation, and rapid functional recovery help distinguish SIMD from other acute cardiomyopathies, such as PPCM and TCM [11,12]. Importantly, infectious myocardial involvement may mimic acute coronary syndromes, as demonstrated in cases such as varicella-associated myopericarditis presenting with electrocardiographic and biochemical features suggestive of acute myocardial infarction despite the absence of obstructive coronary disease [13].

PPCM occurs within the last month of pregnancy or up to five months postpartum and is often associated with severely reduced LVEF, sometimes leading to persistent heart failure [14]. TCM is typically triggered by physical or emotional stress and characterized by apical ballooning on echocardiography [15]. In contrast, SIMD generally shows global rather than regional dysfunction, lacks apical ballooning, and demonstrates rapid improvement after infection control.

In our patient, pneumonia-related sepsis during the third trimester caused reversible global left ventricular dysfunction (LVEF 38%). The absence of apical ballooning, the low likelihood of coronary artery disease based on clinical and echocardiographic findings, and full recovery within weeks supported the diagnosis of SIMD.

Management emphasizes controlling the underlying infection and restoring hemodynamic stability [16]. Early empiric antibiotics, careful fluid management, and vasopressor support (preferably norepinephrine) are linked to better survival rates. In our case, early antibiotic use, conservative fluid therapy, and low-dose norepinephrine led to quick clinical stabilization and full recovery of cardiac function.

## Conclusions

In cases of acute heart failure linked to sepsis during pregnancy, SIMD should always be considered in the differential diagnosis. Early identification, appropriate infection treatment, and prompt hemodynamic support can greatly improve maternal and neonatal outcomes.
